# Facilitators and Barriers to Oral Healthcare for Women and Children with Low Socioeconomic Status in the United States: A Narrative Review

**DOI:** 10.3390/healthcare11162248

**Published:** 2023-08-10

**Authors:** Cristian Lieneck, Erin Connelly, Daryah Ireland, Alexandra Jefferson, Jesikuh Jones, Nicole Breidel

**Affiliations:** 1 School of Health Administration, Texas State University, San Marcos, TX 78666, USA; 2 School of Health Sciences, Southern Illinois University-Carbondale, Carbondale, IL 62901, USA; econnelly@siu.edu (E.C.); daryah.ireland@siu.edu (D.I.); alexandria.jefferson@siu.edu (A.J.); jesikuh.jones@siu.edu (J.J.); nicole.breidel@siu.edu (N.B.)

**Keywords:** oral health, dental care, women and children, socioeconomic status

## Abstract

This rapid review examined facilitators and barriers affecting oral healthcare access and utilization among women and children with a low socioeconomic status (SES) in the United States from 2019 to the present. A comprehensive search was conducted across multiple electronic databases, yielding a total of 30 relevant studies for inclusion. The findings highlight various facilitators that positively impact oral healthcare outcomes, including targeted educational programs, access to non-dental care healthcare services, community-based initiatives, and increased access to affordable oral health services. Conversely, barriers such as financial constraints, lack of access to food program social assistance, access to care difficulties, and limited oral health literacy were identified as major challenges faced by this population. Understanding these facilitators and barriers during the COVID-19 global pandemic can inform the development of tailored interventions and policies aimed at improving oral healthcare outcomes for women and children with a low SES in the United States.

## 1. Introduction

### 1.1. Purpose

Oral healthcare is an essential component of overall health and well-being, playing a vital role in maintaining quality of life [1]. However, access to adequate dental services remains a significant challenge for certain vulnerable populations, particularly women and children with a low socioeconomic status (SES) [1,2,3]. In the United States, despite advances in dental care and public health initiatives, disparities in oral health persist, disproportionately affecting individuals from disadvantaged backgrounds. This study’s objective aims to comprehensively assess the facilitators and barriers to oral healthcare for women and children with a low SES in the United States, spanning the period from 2019 to the present. Understanding the complex factors that influence oral health outcomes in these populations is crucial for developing targeted interventions and policies that can address the disparities and promote equitable access to dental services, specifically as identified in the literature during the global pandemic period in the United States.

Low SES individuals face multiple interrelated challenges that contribute to the barriers in accessing oral healthcare. These challenges include financial constraints, limited dental insurance coverage, transportation issues, language barriers, and a lack of awareness about the importance of oral health [3]. Furthermore, social determinants of health, such as education, employment, housing, and food insecurity, also intersect with oral health disparities, creating a cycle of disadvantage and limited access to care [1,2,3].

However, there have been efforts to overcome these barriers and improve oral healthcare for women and children with low SES in recent years. Innovative initiatives, such as community dental clinics, mobile dental units, school-based dental programs, and telehealth services, have shown promise in expanding access and providing preventive and treatment services to underserved populations. Additionally, advocacy campaigns, public health policies, and collaborations between healthcare providers, policymakers, and community organizations have worked towards reducing disparities and promoting oral health equity.

### 1.2. Contribution to Prior Research

By investigating the most recent (2019 to date) facilitators and barriers of oral healthcare for women and children with a low SES, this article intends to contribute to the ongoing dialogue on oral health disparities and stimulate further research and action. Addressing these disparities requires a multi-faceted approach that encompasses not only healthcare, but also social and economic policies aimed at reducing poverty, improving education, and enhancing access to comprehensive dental services for all individuals, regardless of socioeconomic status. As the COVID-19 transmission rates increased dramatically during the end of 2019, this review addresses or otherwise reconfirms identified constructs in the literature contributing to the oral health status of women and/or children of low socioeconomic status in the United States.

## 2. Materials and Methods

### 2.1. Overview

The research team’s initiative to investigate underlying themes (constructs) surrounding potential facilitators and/or barriers to oral healthcare for women and children with low socioeconomic status from 2019 to the present day. The Southern Illinois University—Carbondale library database (EBSCOhost, Ipswich, MA, USA) research website was utilized to conduct the review and identify publications related to the research topic. Four main databases were queried in the search based upon their overall number of articles identified:

- Academic Search Complete
- CINAHL Plus with Full Text
- SocINDEX with Full Text
- OmniFile with Full Text Select (H.W. Wilson)

### 2.2. Inclusion Criteria

The review team underwent many iterations of search string investigation attempts before identifying an appropriate method that was most inclusive of potential research articles supporting the review topic. Initially, the research team queried the review’s search terms using the Medical Subject Headings (MeSH) website, a controlled vocabulary thesaurus, used for indexing publications in PubMed. The primary search themes in the review (e.g., oral health, socioeconomic status, etc.), however, yielded limited entry terms (lack of exploding vocabulary listings available). Therefore, the research team collectively brainstormed related common terminology surrounding the research topic/theme, to include the use of multiple search engines and collaborative meetings, and ended up using the EBSCOhost’s suggested search terms as auto populated when entering the following primary topics for the review:

- Dental care
- Health literacy
- Access to care
- Socioeconomic status and women

Additionally, the ‘apply related words’, ‘also search within the full text of the articles’, and ‘apply equivalent subjects’ EBSCOhost options were selected to generate additional potential articles for the search. This approach yielded the final search string and Boolean operators utilized for the review, primary based upon the highest number of potential articles identified by the research database:

[(oral health OR oral hygiene OR dental health OR dental care OR oral care OR oral healthcare)] AND [(access to care OR access to oral healthcare OR access to services)] AND [(socioeconomic status OR poverty OR low income) AND (women and children)]

Initial search results were 451 articles and, after filtering for publication dates between 1 January 2019 and 31 December 2023, the research team identified 176 articles.

### 2.3. Exclusion Criteria

Publications were included in the review if all four required topics were addressed in the publication’s main topic, listed as the article’s related words, was identified within the full text of the article by the research database, and/or was listed as an equivalent subject by the research database platform. Publications were included in the review only if they were reported in quality journals (peer-reviewed) and also met the specified publication date range.

**Figure 1:**
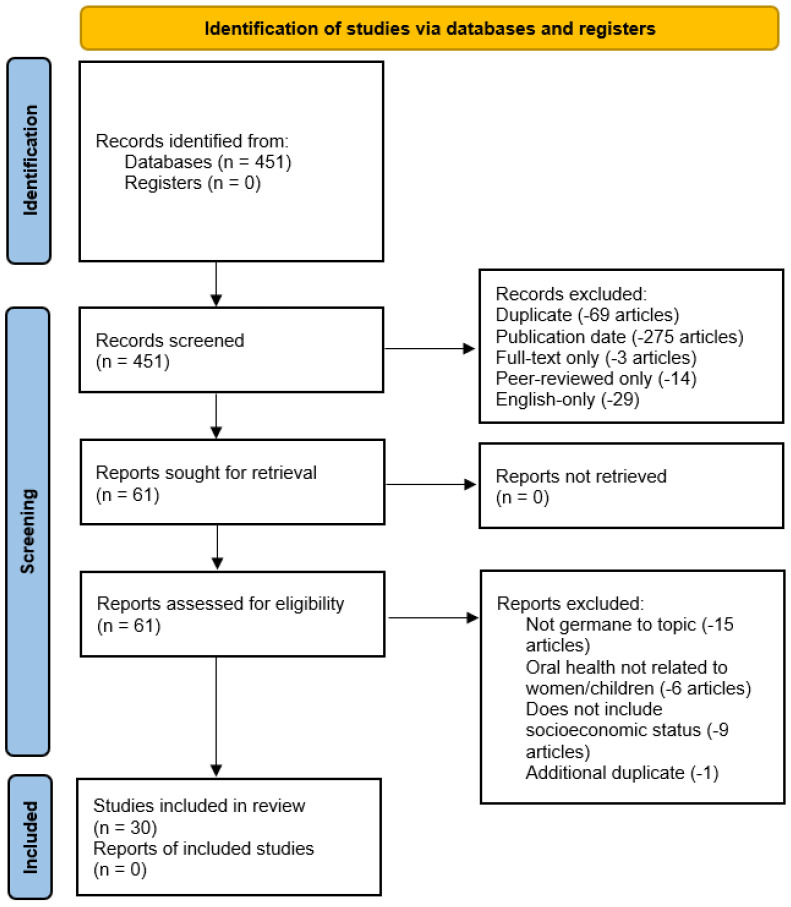
Preferred reporting items for systematic reviews and meta-analysis (PRISMA) figure that demonstrates the study selection process.

Further exclusion parameters were applied by the research team in the EBSCOhost database to identify appropriate publications. In addition to filtering for the publication date, publications that were not available in full-text format, not peer-reviewed, and/or not available in English were excluded. Additionally, only articles published within the United States were included in the review, utilizing the EBSCOhost geography refine checkbox. Figure 1 illustrates the research team’s rapid review process and the applied search exclusion criteria.

A review of the studies included in this review was conducted by the authors, to include a full manuscript review with each identified publication being reviewed by at least two members (or more) of the research team. Table 1 shows the delineation of multiple sets of review articles assigned to the research team members per PRISMA guidelines.

Ongoing research team collaborative meetings via online webinars were continued to progress the review process. The research team’s full text review for eligibility resulted in 61 remaining publications remaining in the review. A total of 31 of the 61 articles were collectively agreed to be removed from the review by the research team, beyond completed automated/database search parameters during the screening process as follows:

- A total of 15 articles were removed for focusing on themes unrelated to this study, yet still mentioning “oral health”. While briefly mentioned, the articles were not addressing oral health specifically but rather just listing it as a healthcare service, along with other unrelated healthcare services.
- Six articles were removed for not being germane to the research topic. While they did mention “oral health” as a healthcare delivery service, “women and/or children” were not addressed in the study.
- Nine articles were removed for also not being germane to the research topic, failing to address “socioeconomic status” in some way.
- One article was identified as an additional duplicate not identified by the research database previously.

The research team continued collaborative, online webinars to address any potential article bias or conflict with the application of the selection criteria for the review. Several consensus meetings resulted in no disagreement among the research team members in this regard.

## 3. Results

### 3.1. Study Characteristics

The team’s full-text review of the 30 articles identified underlying constructs (characteristics) associated with facilitators and barriers of oral healthcare for women and children with a low socioeconomic status within the United States. A summary of review findings for each article is provided in Table 2. Additionally, six literature reviews (systematic, rapid, and/or scoping) were also identified in the final search articles by the database, as noted by an asterisk next to the reference number.

### 3.2. Identification of Underlying Constructs

Identifying underlying themes involved a meticulous and structured approach. The process began with the research team reading and comprehending the included studies within the rapid review. The research team then engaged in a process of data extraction, systematically extracting relevant information from each study, including study characteristics, methodologies employed, and key findings by beginning a sub-group draft completion of Table 1 columns for their assigned review articles. Upon completion, the review team conducted the thematic (construct) process of thematic analysis and identification through live/virtual collaborative meetings and web-based, real-time affinity diagrams and other tools available via webinar software. This iterative process of analysis, interpretation, and synthesis allowed for the identification of underlying themes, contributing to a comprehensive understanding of the research topic. Specifically, reviewers 2 through 6 (from Table 1) presented their 10 assigned articles and initial, key underlying themes identified in the literature to the research team. As the duplication and reoccurrence of themes arose, corresponding team members added the construct(s) to the webinar-hosted affinity diagram. Upon completion, reviewer 1 assisted in the further discussion and collapsing of identified, similar themes across the research team’s discussion.

**Figure 2:**
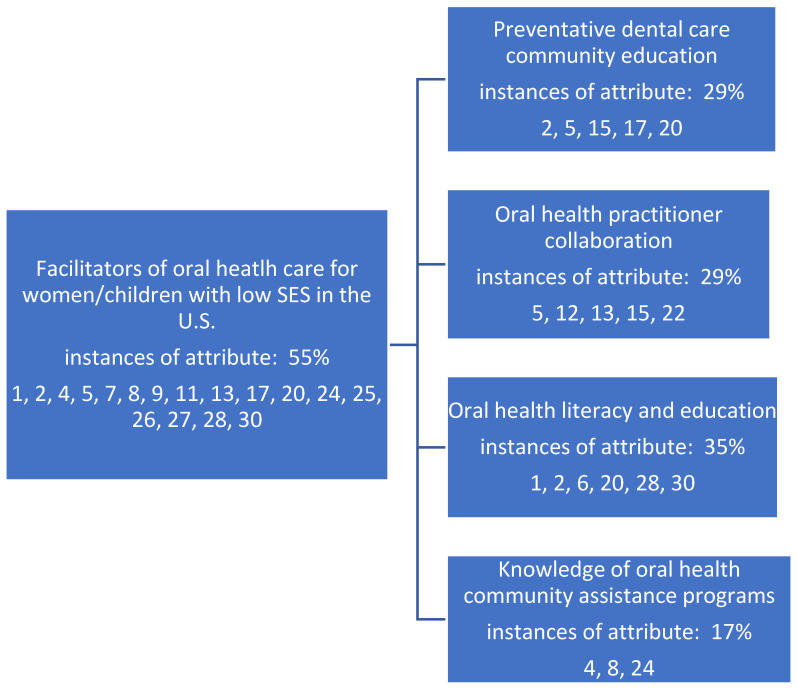
Underlying stakeholder themes (constructs) serving as facilitators as identified in the literature and supporting metadata for the oral health status of women and children of low socioeconomic status in the United States.

The review team’s consensus meetings identified multiple facilitator and barrier themes related to the research topic. Most often, themes were easily identified as facilitator and barrier sub-themes, inversely related. Five themes were identified to support facilitators of oral healthcare in the United States for women and children with low socioeconomic statuses. These underlying themes (constructs) are identified in Figure 2 and the articles supporting these underlying facilitator constructs occurred in 55% of the identified review articles. Findings are not mutually exclusive to either sub-construct identified below, as several articles supported each theme.

**Figure 3:**
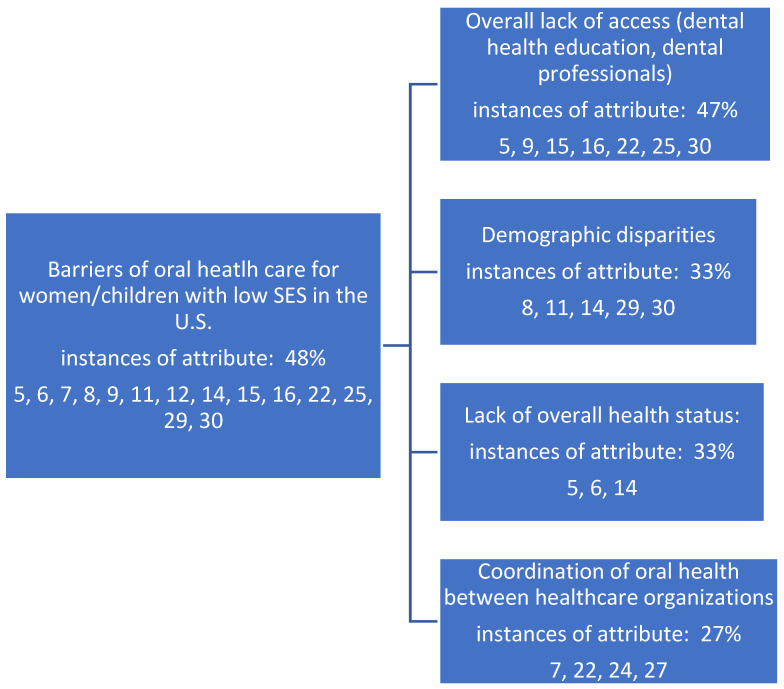
Underlying stakeholder themes (constructs) serving as barriers as identified in the literature and supporting metadata for the oral health status of women and children of low socioeconomic status in the United States.

Further review and analysis supported the investigation into the barriers of oral healthcare upon the specific industry stakeholders focused on for this review. These underlying themes (constructs) are identified in Figure 3, occurring within 48% of the review’s manuscripts. Findings are again not mutually exclusive to either sub-construct identified below, as several articles supported each theme.

## 4. Discussion

Results from this rapid review assist in identifying both facilitators and barriers surrounding the oral health status of women and children in the United States. Often, most identified constructs in the review can be inversely addressed to support the alternative facilitator/barrier theme in the review (either supporting oral health status, or otherwise not supporting oral health status). Therefore, the review team’s assessment of underlying constructs are comprehensively addressed at the overall identified theme level for clarity purposes.

### 4.1. Preventative Education

The promotion of oral health education is vital to reduce the incidence of dental decay and other oral health problems among vulnerable populations, particularly low-income women and children [2]. Preventative dental visits and early dental checkups are crucial for instilling good oral hygiene behaviors and detecting dental problems. However, many low-income families face barriers to dental care, including a lack of access to education, resources, and dental professionals [2,5,20].

Preventative dental visits are critical for detecting dental problems, oral abnormalities, and instilling correct oral hygiene attitudes. In fact, the American Academy of Pediatric Dentistry suggests that children create a dental home by the age of 12 months [5,20]. A dental home consists of comprehensive, coordinated, and continuing dental treatment provided by the patient and dentist [5,15,17]. Early preventative dental checkups can help parents create good oral behaviors for their children, such as feeding practices and oral hygiene routines, to help reduce the incidence of early childhood caries (ECC). Yet, for low-income children, the prevalence of ECC or dental caries is disproportionally higher [1,15,17].

### 4.2. Food Insecurity Challenges

Those with low and extremely low food security had poorer dental/oral health results than those with high food security [6,8,25]. Although the links between food security, socioeconomic position, nutrition practices, and dental health are complex, examining these linkages may provide the insight needed to build successful and lasting nutrition education programs, particularly for children and their parents. The evaluation of one intervention in a low-income setting allowed researchers to investigate the relationship between oral health concerns, income, food security, eating behaviors, and attitudes [8,25]. Concerns about overall health logically follow oral health risks. So, while delivering oral health education and integrating it with a strategy to improve eating competence is encouraged, the findings imply that oral health professionals should pay attention to food security as a significant influence upon oral health status for women and children of low socioeconomic status.

### 4.3. Oral Health and Other Healthcare Practitioner Collaboration

Oral health practitioners are advised to support federal and state food assistance programs and local food banks. Offering meal planning and budgeting advice, ideas to increase dietary variety on a budget, and how to address eating contexts (e.g., eating as a family, turning off screens, food neutral mealtime conversation) to encourage regular meals and feeling relaxed about eating are all practices that address both eating competence and food security [12,15]. Concerns about overall health logically follow oral health concerns. However, the success of an eating approach must address food insecurity, which poses a significant barrier to oral health and, as a result (since overall health concerns logically follow dental health issues), the general health of such vulnerable people.

Many mothers who are under prenatal care still experience barriers to dental care [5,22]. Health education and promotion is something that every physician should promote; this article tells us how women who are receiving prenatal care are not educated on dental care. Being educated in areas that will help to promote dental care amongst women who are pregnant is something that is important and the physicians who are seeing these women may not be aware of such circumstances [22]. Horowitz et al. stated that women who are pregnant that go to the dentist get backlash because of safety reasons, when it is perfectly safe for women to have oral care while pregnant [5,22]. Having a physician who has knowledge about oral health can help with the treatment of patients and it should be an all-around teaching even if you are in a specialty.

### 4.4. Health Literacy and Education

Several studies identified in the review demonstrate the significance of oral health literacy among low-income pregnant women and its impact on access to dental care [1,20,30]. Researchers found that pregnant women with low levels of oral health literacy were less likely to receive regular dental care during their pregnancies, leading to an increased risk of dental problems and adverse pregnancy outcomes [20,30]. Research also identifies the need for oral health education programs targeted to improve oral health literacy among this vulnerable population, ultimately leading to better dental care and overall health outcomes [1,28,30]. By providing educational resources and interventions that address the unique needs of low-income pregnant women, healthcare providers can improve the oral health of both mothers and children. Sullivan et al. highlight the importance of oral health literacy among caregivers of preschool-aged children [2,6]. The authors conducted a systematic review identifying that many caregivers lack the necessary knowledge and skills to promote good oral health practices in young children, leading to an increased risk of tooth decay and other oral health problems. The authors suggest that providing caregivers with the necessary oral health education and resources, such as age-appropriate toothbrushing techniques and healthy food choices, is a critical component to prevent dental problems in young children [2,6]. This underscores the need for targeted oral health education programs for caregivers, which can be delivered through multiple channels, including healthcare providers, early childhood education programs, and community-based organizations.

### 4.5. Knowledge of Oral Health Community Assistance Programs

Joufi et al. examines the role of early childhood education programs in promoting oral health among low-income families in the United States [4,8,24]. The author’s review found that many Early Head Start programs lack sufficient oral health education and resources, leading to disparities in oral health outcomes among low-income children. The study underscores the need for partnerships between early childhood education programs and dental professionals to enhance the delivery of oral health education and services to low-income families. By incorporating oral health promotion activities and education into early childhood education programs, such as providing dental screenings and promoting good oral health habits, early childhood educators fill an important gap in improving oral health outcomes among low-income families [4,8,24]. Ultimately, this can lead to better oral health and overall health outcomes for vulnerable children and families.

### 4.6. Demographic Disparities

There are many racial and ethnic barriers that hinder our healthcare system. In the article “Racial/Ethnic Differences in Oral Health Knowledge and Practices of Preschoolers’ Parents”, a study was completed that revealed preschoolers from low-income homes are more likely to have dental caries (cavities) [8]. Oral healthcare has not been included in the whole of primary care because there is a disconnect that physicians feel they are not able to fully give patients guidance on anything dental. This barrier is one that can be easily solved because the way to prevent dental caries is by eating less sugar or things that have sugar in them. Primary care doctors should monitor the sugar intake that a child is receiving so that they ensure that their patients are healthy [8]. Musselman stated that parents should be educated on their child’s oral health when they are infants, so that they can prevent them from having cavities when they are between the ages of 2–5 years old [8]. When children are taught things at a young age, they are able to incorporate it into their adulthood. Knowing that dental hygiene is important helps with care. Social determinants of health inform us that where we live plays a role in how we receive care.

### 4.7. Access to Care and Other Oral Health Disparities

Lack of access to care, including dental insurance, transportation, and availability of providers, has been linked to inferior oral health outcomes, such as higher rates of dental tooth decay and periodontal disease, in underserved and marginalized communities. The impact of access to care on oral health disparities is particularly significant for low-income individuals, racial and ethnic minorities, and rural populations, who are excessively affected by oral health disparities. According to Attanasi et al., while it is known that children, up to age 18, of families living below the poverty level, are in greater jeopardy than other equally aged associates of developing dental cavities, it is also significant that the apparent threats due to dental ailment, have originated to be low in teenage populations [9]. Studies indicate that intercessions intended at improving access to care, such as community-based curricula and strategy changes, can help diminish oral health disparities by boosting the utilization of preventive services and advocating early exposure and treatment of oral health problems [9]. However, addressing oral health disparities requires a comprehensive approach focusing on social health elements and augmenting health literacy to promote awareness and behavior change.

Factors such as income, race/ethnicity, and geographic location affect access to care and contribute to disparities in oral health. For example, people with lower incomes may be unable to afford preventative dental care, leading to more vast and expensive dental procedures in the future. Improving access to care through programs such as Medicaid and community health centers can help decrease oral health disparities by providing preventive services and preliminary treatment for oral health issues. However, systemic changes are necessary to address the root triggers of oral health disparities and ensure that all individuals have reasonable access to high-quality oral healthcare.

Health intervention programs and community health initiatives aim to promote the change to access to care and health disparities among low-income communities and minorities. The long-term efforts of the intervention efforts are more complex and should aim towards outcomes related to risk behavior and a sense of community rather than health status and life satisfaction. Reaching these communities can be a challenge especially for oral healthcare needs. Community participation plays a role in the success of intervention programs. According to Nickel and Knesebeck, higher participation and engagement levels in community programs results in more positive health outcomes [15]. To reach low-income communities and minorities, many dental and dental hygiene schools have incorporated curricular interventions to reduce health disparities due to race or ethnicity. These interventions aim to promote cultural competency and the understanding of diversity to improve communication and address oral health disparities [15].

### 4.8. Lack of an Individual’s Overall Health Status

Many individuals do not associate oral health with systemic health. This lack of knowledge and lack of awareness can have detrimental effects on a person’s oral health. Education through case management and interprofessional communication will improve awareness for patients oral and overall health needs. Many communities that are underserved lack the ability to obtain routine care for their overall health. Schmidt et al. states that periodontal disease is more prevalent among individuals who live below the federal poverty level [6]. In many rural areas where there is a lack of access to care or higher rates of poverty, there is a higher rate of health conditions and oral health conditions such as periodontal disease. These health and oral health conditions can go untreated due to the lack of education and lack of access to care. Many community-based interventions attempt to reach these individuals to provide the education, awareness, and opportunities for the individual to get the care they need [6].

### 4.9. Care Coordination among Healthcare Organizations

Interprofessional collaboration and care coordination are vital to delivering high-quality patient-centered care in healthcare systems. An interprofessional partnership can improve patient outcomes, reduce healthcare costs, and increase patient satisfaction. Relationships and organization among healthcare professionals can ensure that patients obtain comprehensive, coordinated care, leading to better health outcomes and improved quality of life [27]. Healthcare systems can facilitate interprofessional collaboration and care management by providing resources, such as electronic health records and communication tools, that allow healthcare professionals to share data and organize care efficiently. In addition, education programs emphasizing interprofessional association and care coordination can prepare healthcare specialists to work successfully as part of a team and deliver high-quality patient-centered care. Chari et al. state while it is known that the sociopolitical perspective and the characteristics of an oral healthcare system are forced to affect oral health and oral health inequality, no evaluation has assessed the significance of inequality for clinical oral health statistics [27].

Physicians getting together for the betterment of patients will always be something that will not only improve patient care but also our healthcare system as a whole. Systems have been created that allow different hospitals to connect with one another to share information with colleagues, or there may be some people who want a transfer of care from one place to another. All of these things require physicians to coordinate care with one another, teach each other, and also sharpen skills that may not need to be used on an everyday basis [7,22,24]. Physicians in Haber et al. stated that “in order to improve oral prenatal care collaboration needs to happen so that physicians can be aware as well as the patients on how visiting the dentist while pregnant is important” [7]. Being able to collaborate with peers will help to bring new solutions that can help the betterment of America’s healthcare system as a whole.

Collaboration is something that is always needed in healthcare systems because everyone has to work together to provide the best care for patients. A study was performed on the nurses who visit these new mothers so they would understand if they were mentioning dental hygiene in the overall health overview Haber et al. [7]. Care collaboration is performed when the responsibility of educating a patient is shared by everyone, and from this article the nurses are checking everything. There were a few nurses who did not talk about oral care because they were not sure what to tell the patients (Haber et al.) [7]. Going to the dentist should be carried out regularly, the same as seeing your primary care doctor, and care collaboration can make this possible. If you ask your patient when the last time they saw a dentist, and they say two or three years ago, that is perfect timing to explain how important dental hygiene is for oral health in this stakeholder population.

## 5. Limitations

Despite the valuable insights gained from the systematic literature review, there are several limitations that should be acknowledged. The quality and reliability of the included studies vary, as the review relies on the available published literature within a very specific database search timeframe to meet the researchers’ initial review intent surrounding the global pandemic and rates of transmission. Additionally, the search strategy employed may have inadvertently excluded relevant studies, potentially introducing a selection bias. The search results included six literature reviews of various capacities (systematic, rapid, scoping, etc.), which the research team felt relevant to report and utilize due to the aggressive, required search terminology, publication date range, and Boolean operators.

The review team also identified that a more thorough (systematic) review (beyond extending the publication date range) may also involve expanding studies beyond the United States to perhaps provide additional insights to the currently known facilitators and barriers for access to oral health services for women and children with low socioeconomic status. Expansion of the search dates/parameters would potentially lead to a much more comprehensive review; however, the review team specifically wanted to conduct a rapid review to investigate facilitators specific to the COVID-19 pandemic period. While this is easily viewed as a limitation, the team’s overall intent was to attempt to identify any new facilitators and/or changes to what has already been identified in the previously published literature. As such, the date range for this rapid review was determined a priori. Additional research databases, such as PubMed, could have also been included in the initial search.

## 6. Conclusions

In conclusion, addressing the facilitators and barriers of oral healthcare for women and children with low socioeconomic status is crucial for promoting oral health equity in the United States. By prioritizing comprehensive interventions, engaging in collaborative efforts, and implementing evidence-based policies, it is possible to reduce disparities and ensure that all individuals, regardless of socioeconomic status, have access to the oral healthcare services they need to achieve optimal oral health and overall well-being.

## Figures and Tables

**Table 1:** Reviewer assignment of the initial database search findings (full article review).

Article Assignment	Reviewer 1	Reviewer 2	Reviewer 3	Reviewer 4	Reviewer 5	Reviewer 6
Articles 1–10	X	X	X	X		
Articles 11–20	X	X	X	X	X	X
Articles 21–30	X				X	X

**Table 2:** Summary of included articles in the rapid review (*n* = 30).

Reference Number	Author(s)/Year	Article Title	Purpose/Method	Observation/Outcome
[1]	Maybury et al., 2019	Oral Health Literacy and Dental Care among Low-Income Pregnant Women	The purpose of the article was to see amongst low-income pregnant women in Maryland the impact of oral health literacy and knowledge of oral health. A survey was utilized and one-on-one interviews.	Findings demonstrated that the majority or women lacked knowledge and/or the importance of dental care while pregnant.
[2] *	Sullivan et al., 2022	Oral Health Literacy Inventories for Caregivers of Preschool-aged Children: A systematic review	The article investigated how much the caregivers of preschool-aged students knew about oral health literacy.	Findings demonstrated that there was not much on literacy and knowledge that they knew. Furthermore, there should be more to evaluate the caregiver’s oral health literacy.
[3]	Hardgraves et al., 2020	Attitudes, Expectations, Knowledge, and Intentions Regarding Oral Health: Perceptions of Older Adults	The purpose of this article was to assess health literacy and access to care while including attitudes and intentions related to dental care utilization. The perspectives of older adults living independently were investigated further. A qualitative thematic analysis was guided by behavioral constructs of the Reasoned Action Approach. Results were utilized to develop and conduct semi-structured interviews of a purposeful sample of adults aged 65 and older living independently. A demographic questionnaire, followed by one-on-one interviews was conducted by the primary investigator from August of 2018, through January of 2019.	Findings included perceptions of participants that suggested socioeconomic disparities could be decreased through interprofessional efforts, health literacy education, dental professional curriculum focused on better understanding the complexities of the healthcare system. Additional observations of the reevaluation of policies that limit the ability of dental hygienists to work to the full extent of their scope of practice were also observed.
[4] *	Joufi et al., 2021	Oral Health Education and Promotion Activities by Early Head Start Programs in the United States: A systematic review	The study investigated how to talk about oral health literacy to prevent dental caries amongst children in the early childhood bracket. The method was a quantitative study that was taken from five studies to collect the data.	Findings identify there was positive outcome that reflected oral knowledge and behavioral of practicing the knowledge.
[5]	Horowitz et al., 2019	Obstetric Providers’ Role in Prenatal Oral Health Counseling and Referral	Certified midwives were investigated in Maryland to see how much they refer their patients to dentist and the barriers that are in place for their oral health. A qualitive descriptive study was used to call physicians to one by one to obtain their thoughts on the need for dental care amongst pregnant women.	Results suggest the providers did not know that there was a need for prenatal dental care. Dental care was not something that physicians discussed with their patients or thought that they needed to discuss it, which made them want to include it in their prenatal care.
[6]	Schmidt et al., 2021	Integrating Case Management into the Dental Hygienist’s Role: Improving Access to and Utilization of Oral healthcare for Pregnant Women	The study summarizes the University of Maryland School of Dentistry’s (UMSOD) Dental Hygiene program’s case management protocol for low-income pregnant women in an effort to increase the utilization of oral healthcare services. This program was designed to expand access to oral healthcare by integrating the dental hygiene faculty and students into the prenatal healthcare protocol at a university-based women’s health center. Data were being collected monthly by the program coordinator since program initiation in 2018, to evaluate effectiveness. Measures included the number of pregnant women referred to the UMSOD from the UMWHC, the number of pregnant women who report for dental appointments at the UMSOD, the number of pregnant women who do not show for dental appointments at the UMSOD, and the number of pregnant women who complete comprehensive dental hygiene care.	Results to-date indicate that partnering with the UMWHC and providing case management services caused an increase in referrals, the number of pregnant women who have dental appointments, and decreased the percentage of pregnant women who do not show for appointments. Additionally, there was an increase in the percentage of pregnant women who completed dental hygiene care in the analysis.
[7]	Haber et al., 2020	Promoting Oral Health for Mothers and Children: A Nurse Home Visitor Education Program	The study’s purpose is to look to see if the nurses who were given oral health classes were helping to better their education and teaching positive oral health to patients. A quasi-experimental design was used to detect oral health in an evidence-based care curriculum and the ability to increase oral health practice behavior. This initiative was also studied to see if it increased oral health outcomes for patients. A pre- and post-survey were issued to measure the effectiveness of the training.	The outcome of this study was that it improved as long as the nurses were aware they could include it in their study. The percentage of participants who answered questions was reported. The study concluded that the results showed that the NPs did increase oral health education.
[8]	Mussleman, 2020	Racial/Ethnic Differences in Oral Health Knowledge and Practices of Preschoolers’ Parents	The study explored racial/ethnic similarities and differences in oral health knowledge and practices among parents with pre-school age children. Areas of oral health needing further education were investigated. A descriptive statistics analysis was used with a convenience sample of 116 parents of Hispanic, Asian, and non-Hispanic Black children aged 3 to 5 years attending Head Start centers in San Francisco, California. A quantitative study which used a convenience sample to survey parents of 3–5-year-old children who attended a head start program. The researcher created her own survey using a previously validated survey—SMILE. A descriptive correlational method was utilized to analyze survey results.	The study identified that parents were knowledgeable about and incorporated most aspects of oral hygiene into their daily lives. Gaps between knowledge and practice were also observed. Pediatric nurses were identified as possessing an opportunity to promote children’s oral health by educating parents and providing anticipatory guidance on dental caries prevention when caring for children in schools, clinics, and acute care settings. Results demonstrate that there were statistically significant differences in oral health knowledge between difference racial and ethnic backgrounds. It was concluded that the pediatric nurse may be the best first line of defense in education as almost all respondents’ children had seen a nurse, but not all had seen a dentist or dental professional.
[9]	Attanasi et al., 2020	Preventive Dental Care Programs for Children: Parental perceptions and participation barriers	The study observed an increase in dental literacy and an understanding of the barriers that are not allowing people to have dental care. A qualitive care study that sampled people who did and did not know about the free preventative dental care was utilized.	Results indicate there needs to be different attention to the different cultures and the local residents as well.
[10]	Claiborne et al., 2022	Disparities in Caregiver-Reported Dental Cavities and Toothaches Amo, ng Children in the Special Supplemental Nutrition for Women, Infants, and Children (WIC) Program	Researchers used the 2016 The National Survey of Children’s Health (NSCH), a nationally representative dataset, and examined the disparities in reported tooth aches and cavities in children aged 2–4 years over three categories of WIC eligibility and participation. Data were extracted and a regression model was used to identify the proportion of dental cavities and tooth aches reported between the three groups: eligible WIC and participation, eligible WIC and non-participating, and non-eligible for WIC.	The researchers found that WIC the WIC participating group had more reports of cavities and toothaches in children ages 2–4 and concluded that more oral health education must be provided. The study’s data are 7 years old at publishing and may not be representative of the current population.
[11]	Albright et al., 2020	Oral health among student veterans: Effects on mental and physical health	Research explored differences in oral health in both active-duty service members and veteran students in postsecondary education. Data were collected from 2011 to 2014 using a National College Health Assessment Survey. Five different questions and regression models were used to attempt to control confounders.	It was observed that self-care behaviors related to maintaining the oral health of service men and women in post-secondary education was present when compared their nonservice member peers.
[12]	Lohse & Masters, 2019	Eating Competence and Oral Health in Supplemental Nutrition Assistance Program Eligible Populations	The purpose of this quantitative study was to identify if including an eating approach in oral health education is beneficial. The study’s sample size was very small (96 participants), only included individuals who could read and speak English, and only from Pennsylvania—reducing the power of the study. Interestingly, the researchers listed several limitations, However, sample size was not one of them.	Most of the study respondents were female and while limited, the study does highlight implications between food insecurity and oral health.
[13]	Ludwig et al., 2019	Color-Blind Racial Attitudes in Dental Hygiene Students: A pilot study	The purpose of this article was to determine the color-blind inequalities of dental hygiene students. The study was conducted using first-year and second-year students in a dental hygiene program in Virginia. High scores indicated levels of the denial of racism. While seventy surveys were completed, the majority of them were Caucasian females under the age of thirty.	There was obvious racial unawareness of racial privilege as cited by the manuscript. This article can play a role in showing how there is a racial basis when it comes to dental hygienist students.
[14] *	Gultekin et al., 2020	Health Risks and Outcomes of Homelessness in School-Age Children and Youth: A Scoping Review of the Literature	This study was conducted to determine how children who were homeless had a higher chance of having health issues. Children (ages five to eighteen) who experience homelessness have higher rates of malnutrition and other medical issues were investigated. School age children were studied, as well as runaway youth, and only kids in the United States from 2005 to 2019.	The research identified that preventative services which address the kids’ physical and mental needs are vital to succession in adulthood. This article can correlate oral healthcare in kids, by highlighting the lack of care that homeless kids receive.
[15] *	Nickel & Knesebeck, 2020	Effectiveness of Community-Based Health Promotion Interventions in Urban Areas: A Systematic Review	The study summarized that health campaigning in urban neighborhoods has been a community-based approach in the past decade. Further, large-scale neighborhood established health promotion systems—however, often resulted in inadequate or unaccounted for population-wide variations.	There were no actual persons involved, a systematic literature review was conducted, and findings were eliminated based on whether the material discussed urban areas. This is another article that discusses the inequality of care and can be associated with socioeconomic inequalities.
[16]	Lello et al., 2019	Health Disparities among Children with Autism Spectrum Disorders: Analysis of the National Survey of Children’s Health 2016	The article cites that children with health disparities have more unmet healthcare needs and barriers to access to care. A quantitative statistical analysis was used in this research.	The study’s findings included factors influencing barriers to access to care, factors influencing unmet healthcare needs, and interventions to improve access to care. Public programs and community work were mentioned as interventions to improve the unmet healthcare needs for children with ASD and other health conditions including co morbidities.
[17]	Yamanis et al., 2020	“Hay que seguir en la lucha”: An FQHC’s Community Health Action Approach to Promoting Latinx Immigrants’ Individual and Community Resilience	The manuscript identifies that immigrants have less access to healthcare than non-Hispanics. Despite many barriers that exist for immigrants, they demonstrated resilience for their families in their attempt to access quality healthcare. This was a qualitative study utilizing interviews as primary data collection.	This study found and highlighted the importance of reaching minorities, overcoming barriers to access to care, public policy, resilience, and immigration.
[18]	Thompson et al., 2019	Social Determinants of Health and Human Papillomavirus Vaccination Among Young Adults, National Health Interview Survey 2016	The article addressed that HPV can be found orally. This was a qualitative study through a National Health Interview Survey and identified data related to social detriments for healthcare with an emphasis on HPV include education level, economic status, health literacy, and insurance coverage.	This study’s results give insight to social detriments related to healthcare and separates data for men and women.
[19]	Cecilia, 2020	State-Level Immigration Policy Context and Health: How Are Latinx Immigrant Parents Faring?	A hierarchical regression analysis was used to evaluate perceived immigration policy issues and healthcare. Immigrants face many health issues and access to health. This article explores financial issues, Employment difficulties, fear of disintegration leads to additional stress to families and children.	This study leads to imply that immigrant families need health programs to support their healthcare needs.
[20]	Ge et al., 2019	Classification Tree Analysis of Factors Associated with Oral Cancer Exam	A classification tree analysis was performed by data mining to differentiate factors associated with oral cancer exams.	Research identified unique population subgroups that could be targeted in future tailored public health interventions. Specific to underserved populations, it identified how important it is to develop and implement community-based interventions that encourage regular dental visits and provide oral cancer self-examination education.
[21] *	Berini et al., 2022	Impact of Community Health Workers on Access to Care for Rural Populations in the United States: A Systematic Review	The purpose of this review was to describe CHW interventions and their outcomes in rural populations in the United States.	Interventions by Community Health Workers (CHWs) were identified and demonstrated a reduction in disparities for underrepresented populations. There is a shortage of support for CHW integration in healthcare delivery. This finding may be especially important in rural areas of the United States, where access to care remains a challenge.
[22]	Das et al., 2020	Oral health literacy: A practical strategy towards better oral health status among adult population of Ghaziabad district	The purpose of this study was to investigate the level of oral health literacy and its impact on socioeconomic and oral health status among adults in the Ghaziabad district.	Despite significant efforts by health professionals to promote oral health and develop beautiful smiles, there is undoubtedly a gap between oral health knowledge and practice.
[23] *	Tavousi et al., 2022	Measuring health literacy: A systematic review and bibliometric analysis of instruments from 1993 to 2021	The purpose of this study was to review all existing instruments in order to synthesize current information on the development of existing measurement instruments, as well as their possible translation and validation in languages other than the original languages.	There were more than enough instruments for measuring health literacy present in the literature of today. The review also identified that a number of instruments did not report psychometric properties sufficiently.
[24]	Bastani et al., 2022	How does the dental benefits act encourage Australian families to seek and utilize oral health services?	The purpose of this study was to examine the content of the Dental Benefits Act 2008, which serves as the foundation for the Child Dental Benefits Schedule.	In order to assess how the Act promotes Australian families to seek and use oral healthcare.
[25]	Wilson et al., 2022	Clinical practice guidelines and consensus statements for antenatal oral healthcare: An assessment of their methodological quality and content of recommendations	The study evaluated the content of suggestions in antenatal oral healthcare guidance materials and the quality of their methodology in order to inform areas of development, clinical practice, and research focus. The study identified a total of 98 discreet recommendations were identified and demonstrated considerable unanimity but differed in scope and level of information.	The study included guidance documents and revealed areas of strengths and limitations and posit areas for improvement. Future research on the adaptation of antenatal oral healthcare guidelines and consensus statements to local contexts was recommended. Further, more high-quality studies examining interventions within antenatal oral healthcare are needed to support the development of recommendations.
[26]	Goldfeld et al., 2022	Comparative inequalities in child dental caries across four countries: Examination of international birth cohorts and implications for oral health policy	The goal of this study was to compare the number of disparities in child dental caries among four high-income nations, as well as their policies for child oral health. Data analyses were coordinated among four prospective population-based birth cohorts.	This study suggests that lesser gradients in settings with combinations of universal dental coverage and/or fluoridation were necessary. Further suggestions state that these provisions may ameliorate inequalities through additional benefits for socio-economically disadvantaged groups of children
[27]	Chari et al., 2022	Oral health inequality in Canada, the United States and United Kingdom	The goal of this study was to evaluate the severity of absolute and relative oral health inequality in countries with similar socioeconomic environments. This included different oral healthcare systems, such as Canada, the United States (US), and the United Kingdom (UK), in the first decade of the new millennium.	The study identified significant oral health inequality in all three countries. Among such individuals, inequality in untreated decay was highest among Americans. Inequality for filled teeth was negligible in all three countries. For edentulism, inequality was highest in Canada. Further, there remained a higher concentration of unmet needs among the poor in all three countries.
[28]	Zimmerman & Rodgers, 2022	Exploring Ways of Knowing: Teaching the Skill of Health Literacy to Refugee and Immigrant Women	Thes study cited that after landing in the United States, refugees and immigrants suffer poor health outcomes.	Results indicate that, regardless of the person’s health status at the time of entrance, these bad consequences tend to be disproportionate to those of the general population. Strong health literacy skills, according to research, can improve health outcomes in this subgroup.
[29]	Dudovitz et al., 2020	Improving parent oral health literacy in Head Start programs.	A total of 29 Head Start agencies across the country were trained to deliver a parental oral health literacy intervention. Surveys were conducted to assess measured parent and child demographics, oral health knowledge, behaviors, information sources, and healthcare utilization.	The intervention increased parental oral health literacy, enhanced parental oral health engagement, improved child oral health behaviors, and facilitated health communication with parents.
[30]	Claiborne & Poston, 2020	Innovative Collaborative Service-Learning Experience among Dental Hygiene and Nurse Practitioner Students: A pediatric oral health pilot study	The study piloted an innovative, collaborative service-learning (ICSL) experience for dental hygiene (DH) and primary care nurse practitioner (NP) students to address pediatric oral health.	There was a positive change in interprofessional socialization scales for all study participants. The study’s experience had a positive impact on NP and DH students’ socialization to interprofessional collaboration to further quality of care.

* Literature reviews identified in the database search.

## Data Availability

Not applicable.
